# A multicentre randomized controlled follow-up study of the effects of the underwater traction therapy in chronic low back pain

**DOI:** 10.1007/s00484-020-01919-8

**Published:** 2020-05-02

**Authors:** Tamás Gáti, Éva Czímer, Györgyi Cserháti, Judit Fehér, Mihály Oláh, Ágota Kulisch, Zsuzsanna Mándó, Tamás Bender

**Affiliations:** 1 Rheumatology Department, Polyclinic of The Hospitaller Brothers of St John of God, Árpád fejedelem út 7, Budapest, 1027 Hungary; 2 Aquarius Experience Bath, Sóstó, Fürdőház tér 2, Nyíregyháza, 4431 Hungary; 3 Medical Department of Bath, Kenézy Gyula University Hospital, Debrecen, Hungary; 4 Hungarospa Hajdúszoboszló Private Limited Company, Hajdúszoboszló, Hungary; 5 St. Andrew Hospital for Rheumatic Diseases, Hévíz, Hungary

**Keywords:** Chronic low back pain, Controlled, Randomized trial, Traction therapy, Underwater therapy, Balneotherapy

## Abstract

Low back pain (LBP) is one of the most costly diseases in the developed world. This study aimed to investigate the effects of underwater traction therapy on chronic low back pain. The primary objective was to prove that underwater traction therapy has favorable effects on LBP. Our secondary objective was to evaluate whether it also leads to improvement in the quality of life. This is a prospective, multicenter, follow-up study. A total of 176 patients with more than 3 months of low back pain enrolled from outpatient clinics were randomized into three groups: underwater weight bath traction therapy and non-steroidal anti-inflammatory drugs (NSAIDs); weight bath; and only NSAIDs. The following parameters were measured before, right after, and 9 weeks after the 3-week therapy: levels of low back pain in rest and during activity were tested using the visual analogue scale (VAS), the Oswestry Low Back Disability Questionnaire, and the EuroQol-5D-5L Questionnaire.

The VAS levels improved significantly (*p* < 0.05) in both underwater weight bath traction therapy groups by the end of the treatment, whereas the improvement in the third group was not statistically significant. Furthermore, the improvements measured in the groups receiving traction therapy were persistent during the follow-up period. There were no significant changes in the Oswestry Index or the EuroQol-5D-5L without VAS parameters in any of the groups.

Based on our results, for patients suffering from LBP pain who underwent underwater weight bath traction therapy, there were favorable impacts on the pain levels at rest or during activity. Clinical trial registration ID: NCT03488498, April 5, 2018

## Introduction

Low back pain (LBP) is one of the most costly diseases due to its high prevalence level that continuously increases parallel to the aging of the population in the developed world. Based on 165 studies in 54 systemic reviews in 54 countries, its prevalence was estimated to be around 12% of the populations on average, between 1980 and 2009 (Hoy et al. 2012). These values also depended on age and sociological status; the point prevalence and lifetime prevalence could reach 79.2% (Kent and Keating 2005). Non-specific lumbar pain is defined as lumbar pain without any known pathological lesions (e.g., tumor, infection, osteoporosis inflammatory disorder, radicular syndrome, fracture, or cauda equina syndrome) (van Tulder et al. 2002). Trials have shown that the possibility of recurrence of low back pain can range up to 44–78% (Airaksinen et al. 2006).

The range for the first line of defense for therapeutic options for chronic lumbar region pain, based on the existing evidence, is as follows: education, home exercises, self-management physiotherapy, balneotherapy, and multidisciplinary pain management. Other therapeutic options that could have positive effects as an addition to the aforementioned treatments are mineral-rich mud compresses, drug therapies (NSAIDs, weak opioids, and muscle relaxants), behavioral therapy, spine schools, mobilization and manipulation, acupuncture and massage therapies, noradrenergic treatment with serotoninergic antidepressants, and capsaicin patch (van Tulder et al. 2003; Abu-Shakra et al. 2014). There are already promising strategies on how to classify the non-specific lower back pain (NSLBP) not yet widespread (Dewitte et al. 2018).

More and more studies seem to be showing that, thanks to the wide range of therapeutic options for LBP patients, surgical intervention has become unavoidable in just certain cases where patients have “red flag” symptoms, which suggest a potentially serious underlying ailment.

With regard to balneo- and hydrotherapy, for the past few decades, evidence-based studies have overtaken simple and unempirical experience and suggest that these therapies actually lead to statistically significant improvement in patients’ conditions (Karagülle and Karagülle 2015).

Also, a number of studies have been done to assess the effectiveness of the different types of traction therapies (e.g., manual, auto-traction, gravitational, aquatic, and mechanical traction) on back pains, but the evidence is not yet clear as to which kind of therapy is recommended to whom and when. For example, there are questions as to whether mechanical lumbar traction should be recommended in combination with other treatments or alone, and under which conditions (Thackeray et al. 2016).

In 2012, Dr. Prasad and his colleagues proved that in a small number of those patients on waiting lists for discus hernia surgery, 77% of them who received combined traction and physical therapy did not require surgery (Prasad et al. 2012).

Another study showed that land-based therapeutic exercise in chronic LBP with nerve compression symptoms are not so effective in pain reduction if the patient first receives aquatic traction therapy (Simmerman et al. 2011)*.*

In 2006, a study that included 24 randomized controlled trials (RCT) assessed the effectiveness of traction in LBP management and found that in mixed groups of patients with LBP with and without sciatica, traction therapy cannot be recommended (Clarke et al. 2006).

Two big sample surveys—one in the UK and the other in the USA—showed that various traction delivery modes were used in 41–76.6% of the cases in low back pain therapy (Harte et al. 2005; Madson and Hollman 2015).

## Aim of the study

The aim of our study was to examine the effect of underwater traction therapy on chronic low back pain.

The primary objective was to measure the hypothesis that underwater traction therapy has favorable effects on LBP by using adjustments to the therapy based on pain parameters. Our secondary aim was to analyze whether this treatment method could result in an improvement in the quality of life.

## Methods

### Study design

In this controlled follow-up of multicentre randomized comparative study, we have analyzed the effects of underwater weight bath traction therapy on chronic low back pain.

We used regular outpatient care clinics to recruit patients. We randomly created three groups. Our study protocol followed the principles in the Helsinki declaration. The study participants read and signed the package leaflet and the consent statement before starting the trial. This study was approved by the Semmelweis University Regional Scientific and Research Ethics Committee (SE TUKEB) (SE TUKEB Number: Number: 21396—3/2017/EKU, Clinical trial registration ID: NCT03488498). The study was also approved by the Institutional Research Committees.

### Participants

Patients suffering from low back pain were selected into three groups at random: receiving a combination of the NSAID medication and underwater traction therapy either traction therapy or only NSAID.

Participants were selected from patients in the Polyclinic of The Hospitaller Brothers of St John of God, the Aquarius Experience Bath in Sóstó, the Kenézy Gyula University Hospital Medical Department of Bath, the Hungarospa, and St. Andrew Hospital for Rheumatic Diseases in Hévíz.

Enrollment criteria were as follows: outpatients aged 18–85 with non-specific low back pain that persists for at least 12 weeks, showing degenerative symptoms, and suffering from moderately reduced mobility. Patient’s pain intensity during activity should have been a minimum of 30 mm on the visual analogue scale (0–100 mm VAS).

Written information on the methodology and process they would be undergoing was provided to each participant, and an informed consent form was subsequently signed before the study. A two-way lumbal spinal X-ray taken within a year was required to be presented.

Exclusion criteria were the following: osteoporotic vertebral compression fractures, severe spondylolisthesis (grade 2 or above), malignancy, pain due to inflammatory spinal disease, severe neurological deficit associated with the lower back, general contraindications to balneotherapy: decompensated cardiopulmonary status, unbalanced endocrinological disease, urine and stool incontinent, infectious disease, fever condition, extensive inflammation/injury/absence of the skin, other severe interstitial and urogenital diseases, decompensated psychosis and neurosis, pregnancy, unconsciousness, and lack of compliance.

### Intervention

Patients were exposed to indifferent water (33–35 °C) for 15–20 min. At the different clinical centers, different components thermal – mineral waters were used but smooth tap water was not used in any of the pools. They were dipped in the water to the neck while they could not reach the bottom of the pool with their feet. During bilateral armpit support suspension, both sides of the ankles had 3–3 kg (kg) weights attached.

Fifteen weight bath therapy sessions were administered during the 3-week period. The duration of the first session was 15 min; this was extended to 20 min from the second occasion.

The doctor met patients three times: first, right before the treatment was started; second, straight after the underwater traction therapy treatments; and third, 9 weeks after the treatment was completed (i.e., 12 weeks after the start of the treatment).

The participants were randomly selected and randomly put into three groups: (1) underwater weight bath traction therapy and non-steroidal anti-inflammatory drugs (NSAIDs) medication, (2) underwater weight bath traction therapy, and (3) only non-steroidal anti-inflammatory drug (NSAID) medication in therapeutic dose. The control group did not receive traction therapy. Throughout the investigation, all participants received their everyday medications. (Participation at physical therapy was allowed for ethical considerations, such as transcutaneous electrical nerve stimulation (TENS) treatments and massage, with these, if any, being documented).

### Outcome parameters

On a visual analogue scale (VAS), patients indicated degrees of pain—both at rest and separately during activity—on a scale from 0 to 100 mm for the past week before the visit. VAS scores were expressed in millimeters (0 = no pain; 100 = excruciating pain).

Functional disability was assessed by using the Oswestry Disability Index (ODI), a self-reported questionnaire which measures the patients’ perceived level of disability in 10 everyday activities (e.g., pain intensity, the changing status of pain, personal hygiene, lifting, walking, sitting, standing, sleeping, social activity, and travelling). The patients scored between 0 and 5 for each of the 10 questions leading to a total score between 0 and 50 that is then expressed in percentage. This questionnaire is validated and has reliability in Hungary (Valasek et al. 2013).

The Hungarian form of the specific standardized EuroQol Five Dimensions Questionnaire (EQ-5D-5L) was used to assess the quality of life of the participants. This self-administered questionnaire is an accepted, and widely used, standardized instrument for evaluating general health status. This system is composed of five dimensions: mobility, self-care, usual activities, pain/discomfort, and anxiety/depression. Participants choose from a scale of 1 to 5 based on the level of difficulty they encounter during such situation (no problem, slight problem, moderate problem, severe problem, and extreme problem). Answers along each dimension are rated as a 1-digit number that is combined into a 5-digit number to create an overall score which describes the patient’s generic health state. The EQ-5D-5L also included an EQ-VAS scale of 0–100, where respondents rated their general health status (0 being the worst and 100 being the best possible health status) (Whynes and TOMBOLA Group 2008). Furthermore, during the visits, checking the criteria for inclusion/exclusion and recording the possible side effects were performed.

### Sample size

The required sample size per group based on the precalculation test with a power of 80% was 32 persons.

Power test based on VAS during activity values was measured at visit II (Springate 2012; Kim 2013).

### Randomization

The statistical processing of the data was carried out by an independent person. The study was single blinded: The statistician had received the anonymous information by e-mail. The groups were created to be homogeneous by age by the statistician. Patients were examined by independent examiners at each visit. The surveys (VAS scales of low back pain at rest and during activity and the Oswestry and EuroQol-5D-5L) were self-administered. The randomization was done by an independent person based on a pre-set system. Size of the group receiving only traction therapy was intentionally set to be double that of the other two to make statistical analyses more reliable.

### Statistical methods

Statistical processing was done using the IBM SPSS 25 software system.

The dataset was first cleaned from missing values (Table 1).

**Table 1:** Summary of the statistical test results

	Eliminated	Total *n*	*n* of analysis	GGeps	Groups		
					1	2	3
VAS activity	14	176	162	0.62	42	81	39
EQ-5D-5L	25	176	151	0.65	38	80	33
EQ-5D-5L-VAS	31	176	145	0.67	35	74	36
VAS relax	16	176	160	0.62	42	82	36
OSWESTRY	24	176	152	0.69	39	78	35

To detect the improvement of the patients, we calculated the differences between the later and earlier values of the variables.

To test the statistical differences of the improvement in the three groups, we ran a one-way repeated measures ANOVA model. We used degrees of freedom correction by Greenhouse-Geisser epsilon (GGeps) to manage the violation of sphericity (*ε* > 0.62). Normality of the residuals was accepted based on d’Agostino’s normality test, and to separate homogeneous groups, Tukey’s post hoc test was run.

Statistical significance was set at the 0.05 probability level for all tests and is expressed as *p* ≤ 0.05 (*), as *p* ≤ 0.01 (**), or as *p* ≤ 0.001 (***). For the per-protocol analysis, missing values were not replaced and were missed from the calculation.

## Results

From June 2017 to January 2019, patient selection and randomization were ongoing. Participants were aged between 18 and 85 years with more than 3 months of low back pain and selected from outpatient clinics.

Patients participated in three visits for the first time before the study, right after the underwater traction therapy treatments, and 12 weeks after the first visit, after completion of the therapy.

The three groups were comparable in terms of age and baseline clinical characteristics. For the groups where it was indicated that NSAID medications were administered, the doses were provided at a therapeutic level.

Due to the randomization process, the distribution of patients per study arm was imbalanced, which resulted in the following group allocations: group 1 = 43, group 2 = 90, group 3 = 43 patients enrolled in the study.

**Fig. 1:**
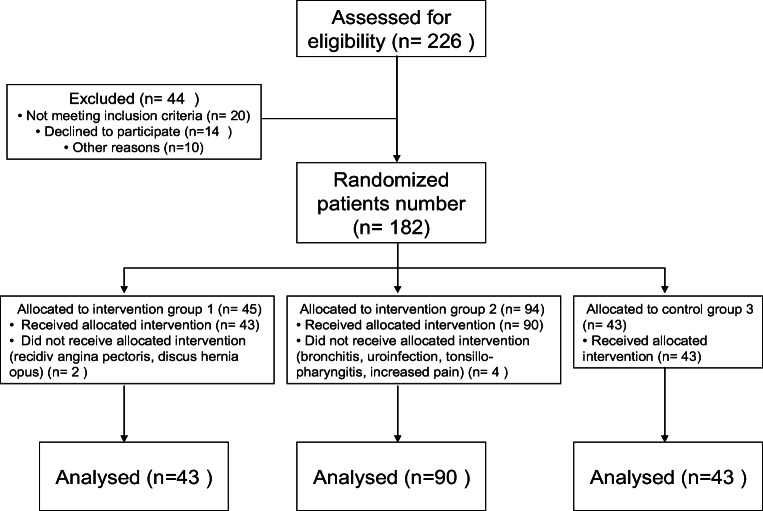
Flow diagram of participants

A total of 226 patients were recruited for the study, and 176 were included in the data analysis. Figure 1 shows 2 patients who were not able to complete the weight bath treatment in the first group (one of them incurred angina pectoris and was excluded from the study and another who had discus hernia opus developed worsening symptoms before the treatment).

Four patients were not able to complete the weight bath treatment in the second group for various reasons that included the following: bronchitis, uroinfection, tonsillopharyngitis, increased pain in the back spine.

In the third group, all of the patients were able to complete the study, although 3 of them requested and received TENS supplemental therapy.

The demographics characteristics are summarized in Table 2.

**Table 2:** Summary of the demographic characteristics

Groups	Age (years), mean (SD)	Gender (*n*)	
		Male	Female
1. NSAID and underwater traction	58.65 ± 12.83	17	26
2. Underwater traction	61.28 ± 11.01	40	50
3. NSAID /Control/	55.14 ± 13.83	13	30

The mean age in group 1 was 58.65 years, in group 2 61.28 years, and in group 3 55.14 years.

### Outcome measures

The study endpoints were to assess differences in pain levels in the visual analogue scale.

The primary endpoint was to determine treatment effectiveness after 3 months following the underwater weight bath traction therapy.

### Statistical analyses

The VAS values at rest of the chronic low back pain patients decreased significantly in the groups treated with underwater traction therapy by the end of the treatment period compared to the baseline (*p* < 0.05); this improvement was observed as well at the follow-up in visit III. There was no significant change in this value in the control group (group 3) where patients received only NSAID medication for chronic low back pain.

While there were no significant differences in the VAS values between the three groups at the time of the first visit, by visit II and visit III, the differences in the VAS values between group 1 and group 3 as well as in group 2 and group 3 became significant (Table 3).

**Table 3:** Results of the statistical analyses

Visits		Group effect	Groups (mean, SD)		
			1	2	3
VAS relax	I–II	*F*(2;157) = 4.38*	*n* = 42	*n* = 82	*n* = 36
			− 25.14 ± 22.76	− 23.41 ± 23.01	− 11.8 ± 13.86
	II–III		1.09 ± 16.84	− 1.43 ± 14.48	− 1.56 ± 15.8
	I–III		− 24.05 ± 19.84	− 24.84 ± 21.8	− 13.36 ± 15.64
VAS level during activity	I–II	*F*(2;159) = 9.44***	− 29.48 ± 24.8	− 29.32 ± 22.12	− 11.9 ± 14.04
	II–III		− 1.00 ± 18.66	− 1.19 ± 18.78	− 1.77 ± 17.24
	I–III		− 30.48 ± 23.27	− 30.51 ± 20.23	− 13.67 ± 20.32
EQ-5D-5L	I–II	*F*(2;148) = 2.94 ns	0.14 ± 0.19	0.14 ± 0.18	0.07 ± 0.15
	II–III		0.01 ± 0.10	0.01 ± 0.09	− 0.01 ± 0.13
	I–III		0.15 ± 0.19	0.15 ± 0.18	0.07 ± 0.2
EQ-5D-5L-VAS	I–II	*F*(2;144) = 6.47**	15.77 ± 18.39	18.41 ± 16.44	7.03 ± 15.26
	II-III		3.63 ± 10.84	2.53 ± 11.11	0.86 ± 8.71
	I-III		19.40 ± 18.53	20.93 ± 19.17	7.89 ± 15.01
Oswestry	I–II	*F*(2;149) = 1.99 ns	− 0.14 ± 0.14	− 0.11 ± 0.12	− 0.10 ± 0.11
	II–III		0.00 ± 0.10	− 0.02 ± 0.08	0.01 ± 0.08
	I–III		− 0.14 ± 0.15	− 0.14 ± 0.14	− 0.08 ± 0.11

The VAS values for lumbar pain during activity also significantly decreased in the groups treated with underwater traction therapy by the end of the treatment compared with the initial stage (*p* < 0.001), and these improvements were also observed in visit III. There were no significant changes in the VAS values in group 3. The differences between the two groups (the underwater traction therapy groups) and the NSAID medication group were found to be significant during visit II as well as visit III (Table 3).

The Oswestry functional disability index change was not significant between the visits in any of the three groups (Table 3). The EuroQol-5D-5L quality of life index change was not significant between either of the visits in any of the three groups.

The EuroQol-VAS change showed that the current general health status also improved in the underwater traction therapy groups (*p* < 0.01) while there were no changes in group 3. The differences between the groups were significant during visit II and visit III (Table 3).

Only patients in group 3 did require extra NSAIDs, opioids, muscle relaxants, or paracetamols for low back pain during the study period.

.

## Discussion

Nowadays, more and more protocols and recommendations appear regarding the treatment of chronic non-specific low back pain. The lumbar spine is the most stressed segment of the spine, where lesions and pain develop most often occur. Non-specific low back pain is also a major public health issue in the world.

The lifetime prevalence of low back pain could reach 38.9% (Hoy et al. 2012) It is estimated that about 11–12% of the total population suffers from a disability and functional decline due to low back pain (Airaksinen et al. 2006).

While we were conducting our studies using modern and standardized methods and data, we also searched for a treatment option that has not yet been analyzed in a large number of randomized trials, which lead us to investigate the impact of underwater traction therapy on LBP. The origins of traction therapy date back to the time of Hippocrates, who used the Hippocratic ladder for traction. Gallenus applied axial stretching for spinal distortions as part of his therapy. In Hungary, underwater traction therapy has a tradition history of about 60 years.

As of now, only a few studies in different traction therapy fields have been run. Current theories regarding its actual physiologic effects indicate that it acutely decreases lumbar lordosis while it concomitantly increases the intervertebral disc height (Pellecchia 1994).

Land-based traction therapies have shown uncertain results, such as form motorized lumbar traction, supine traction, and gravitational traction procedure (Clarke et al. 2006; Macario and Pergolizzi 2006). Nevertheless, these weight tractions also increase tension on the posterior longitudinal ligament that increases the force that has been suggested to temporarily reduce the central, posterior displacement of bulging or herniated intervertebral discs and decreases the symptoms (Ozturk et al. 2006; Unlu et al. 2008).

Blood supply to vertebral bodies may improve during traction therapy, which will enhance the primary source of perfusion from vertebral bodies (Boos et al. 2002).

The effect of traction therapy of the lumbar spine was examined with an MRI in a middle-aged population that showed that traction may significantly improve fluid flow, for at least a short-term, which in turn may influence nutritional inflow and waste product outflow within the matrix of the intervertebral discs (Mitchell et al. 2017).

Meanwhile, in small sample size, weight bath traction hydrotherapy study using controlled lumbar MRI did not find detectable anatomical improvements after the treatments, but the lumbar pain intensity did improve (Oláh et al. 2008).

However, if we studied the overall impact of swimming, it would most probably be evident that while swimming has beneficial effects on muscles and the spine, in general, because muscles actively engaged in swimming contract, the stretching in the spine is less effective than in an inactive, relaxed position during hydrotraction suspension. Simmerman et al. showed in a crossover trial with 30 participants that the aquatic vertical traction results in short-term improvements of the low back pain (Simmerman et al. 2011).

An elongation of lumbal segments (next to each spinous processes) was reported in an underwater traction trial using a subaqual ultrasound measuring method that found that as age progresses, the extensibility of spinal segments decreases (Kurutz 2006a, b). The report showed that after the age of 35 the elongation capacity decreases with aging (Kurutz 2006b).

In our multicenter randomized study, we proved that underwater traction therapy has its place in the physio-, balneotherapy palette.

It has been shown that traction treatment results in long-term healing effects with minimal risk and low cost of intervention. In our findings, the decline in the VAS scale of pain in rest or during activity of LBP patients and the change in the EQ-5D-5L VAS values were significant in those patient groups that underwent traction therapy, proving the improvement in pain sensitivity. However, the Oswestry and the long-term EQ-5D-5L index remained unchanged—as these indexes might have lower sensitivity to change in patients’ pain level—indicating that education and guided physiotherapy may additionally be required to improve quality of life. Furthermore, analysis showed that the NSAID medications were not efficient in improving the chronic low back pain that confirmed the results of several earlier investigations.

A study involving a large number of participants investigated the effects of NSAIDs on chronic non-specific low back pain and found it to be minimally significant in terms of pain reduction. After analyzing the Cochrane overview of 13 clinical trials, there was only a low level of evidence regarding the pain-reducing effects of NSAIDs (Enthoven et al. 2016). A larger meta-analysis reviewing the period between 2007 and 2015 looked at the effects of drug treatments for acute and chronic lower back pain and found that NSAIDs had fewer benefits in chronic lower back pain than previously observed (Chou et al. 2017).

### Limitations of the study

The limitations of this study were the difficulties in blinding the control group due to the nature of the therapy.

The number of participants per each group was not identical; a bias possibly resulted from the multicenter selection. The disadvantage of paper-based questionnaires is that missing data does not immediately appear; thus, it is difficult to recover in the future.

To confirm our findings, more follow-up studies will be required.

The customization of hanging weights based on patient parameters could also increase the efficiency of underwater traction therapy.

## Conclusion

The underwater weight bath therapy is a conservative and easily accessible treatment method for the treatment of low back pain. Based on our results, for patients suffering from chronic low back pain, underwater weight bath traction therapies have a favorable impact on the pain level at rest as well as during activity.
